# Quality of life outcomes and patient-reported experiences in a randomised controlled trial for rifampicin-resistant TB (PRACTECAL-PRO): a mixed-methods study

**DOI:** 10.5588/ijtldopen.25.0823

**Published:** 2026-05-11

**Authors:** K. Lowton, B. Stringer, M. Cusinato, K. Fielding, I. Liverko, R. Moodliar, T.Z. Nikolaevna, V. Solodovnikova, E. Kazounis, B.-T. Nyang’wa

**Affiliations:** 1 Department of Sociology and Criminology, University of Sussex, Brighton, UK;; 2 Manson Unit, Médecins Sans Frontières, London, UK;; 3 TB centre, London School of Hygiene and Tropical Medicine, London, UK;; 4 Republican Specialized Scientific Research Medical Centre of Phisiology and Pulmonology (TB Institute), Tashkent, Uzbekistan;; 5 THINK (TB&HIV Investigative Network), Doris Goodwin Hospital, Pietermaritzburg and Hillcrest, Durban, South Africa;; 6 Nukus TB Hospital and Out-Patient Department, Nukus, Uzbekistan;; 7 Minsk Republican Scientific and Practical Centre of Pulmonology and Tuberculosis, Minsk, Belarus;; 8 Public Health Department, Médecins Sans Frontières, Amsterdam, the Netherlands;; 9 Clinical Research Department, London School of Hygiene and Tropical Medicine, London, UK.

**Keywords:** tuberculosis, drug-resistant TB, RR-TB, QoL, patient-reported outcomes, patient experiences, clinical trial

## Abstract

**BACKGROUND:**

The TB-PRACTECAL clinical trial assessed 24-week, all-oral bedaquiline, pretomanid, and linezolid (BPaL)-based regimens for rifampicin-resistant TB against standard of care (SoC). The PRACTECAL-PRO sub-study evaluated changes in quality of life (QoL), among patients receiving investigational regimens versus SoC, and assessed patient experiences of the new treatments.

**METHODS:**

We recruited participants from Belarus, Uzbekistan, and South Africa. Changes in QoL from baseline to 48 weeks were evaluated using the Short Form-12 (SF-12) and the St George’s Respiratory Questionnaire (SGRQ). Longitudinal analysis was conducted for all six domains of SF-12 and SGRQ. In-depth interviews provided narratives about patients’ experiences.

**RESULTS:**

137 patients participated in PRACTICAL-PRO. All trial arms showed improved SF-12 and SGRQ scores over 48 weeks. Proportional reduction in scores for SGRQ per month was higher in investigational than SoC groups’ Total score (13% vs. 4%), Impact (12% vs. 2%), Activity (14% vs. 7%), and Symptom (15% vs. 4%) domains, indicating quicker QoL improvement. Narratives indicated early treatment satisfaction and social acceptance, with wellbeing relying on family support, clinical contact, and counselling.

**CONCLUSION:**

Patients receiving short, all-oral BPaL-based regimens show improvements across all measured QoL domains, a positive impact on personal relationships, and a return to productive life. TB trials should routinely include patient-reported outcomes.

Effective treatments for drug-resistant TB (DR-TB) are important not only to improve patients’ health but also to reduce community transmission^1^ and antimicrobial resistance.^2^ Uptake of TB care is hindered by psychological and socio-economic barriers,^3,4^ discrimination and stigma,^5^ pill burden,^6^ and health system factors such as knowledge and attitude of health workers.^7^ Historically, management of multidrug-resistant TB (MDR-TB) has focused on treatment adherence and providing affordable and effective care, with outcomes being assessed using clinical markers for improvement such as negative sputum smears and weight gain. More recently, supporting patient wellbeing, particularly quality of life (QoL), has become of increasing interest.^8^ TB affects both physical health and psychological wellbeing,^9^ and there is growing recognition of the importance of understanding the perspectives and experiences of people undergoing treatment. Several generic instruments are available to assess QoL in patients with pulmonary and extra-pulmonary TB,^10^ yet patient perspectives are rarely included in clinical trials despite the complex and dynamic course of patient tolerability of treatment.

Historically, rifampicin-resistant TB (RR-TB) treatment required long (9–20-month) regimens, often with injectables, substantial adverse effects, and modest success rates of 59% in 2018 and 68% in 2021.^11^ In May 2022, the WHO approved a novel oral-only 6-month treatment regimen for programmatic use for most adolescents and adults with RR-TB.^12^ The new guidelines recommend treating most forms of DR-TB with either bedaquiline, pretomanid, linezolid, and moxifloxacin (BPaLM) or bedaquiline, pretomanid, and linezolid (BPaL).^13^ One of the studies underpinning these recommendations was the TB-PRACTECAL trial, which compared three BPaL-based regimens with WHO-approved local standard of care (SoC) in Belarus, South Africa, and Uzbekistan.^14^

To address the limited evidence on how novel new regimens influence patients’ QoL and experiences, we conducted the TB-PRACTECAL-PRO sub-study.

## METHODS

PRACTECAL-PRO (NCT03942354) was a sub-study of the TB-PRACTECAL (NCT02589782)^15^ and used a mixed-methods design involving validated QoL surveys and in-depth interviews in Belarus, South Africa, and Uzbekistan (Supplementary Data). QoL was assessed using the St George’s Respiratory Questionnaire (SGRQ) and the health Short Form-12 (SF-12). The SGRQ has 50 items scored 0–100, with higher scores indicating worse respiratory health,^16^ across three domains: ‘symptoms’, ‘activity’, and ‘impact’, as well as a ‘total score’. The SF-12 is a generic QoL questionnaire derived from the SF-36, containing two domains: ‘mental component summary’ and ‘physical component summary’.^17^ In-depth interviews provided a thorough account of trial-related experiences.

### Participants

PRACTECAL-PRO included TB-PRACTECAL participants with RR-TB aged 15 years or older randomised to BPaL alone (BPaL arm), with moxifloxacin (BPaLM arm), with clofazimine (BPaLC arm), or to the locally approved SoC for RR-TB.^14^ To establish baseline QoL, local healthy volunteers aged 15 years or older were recruited and matched to trial participants (in both investigational and SoC arms) by site, sex, and age (see Supplementary Data). From January 2018 to February 2021, TB-PRACTECAL trial participants were invited to participate in PRACTECAL-PRO at their baseline visit. However, the early interruption of the TB-PRACTECAL trial, the COVID-19 pandemic, and civil unrest at some of the study sites led to some participants being recruited after their baseline visit. For the qualitative component, we purposively sampled participants from the investigational groups based on baseline QoL scores: patients with very poor, midrange, and very high QoL scores were selected, keeping a balance of men and women as well as a range of ages across study sites.

### Data collection

Participants completed QoL surveys at baseline (post-randomisation but before treatment initiation) and at 12, 24, and 48 weeks. Participants’ characteristics were collected at the baseline visit (i.e., age, sex, and HIV status). Healthy volunteers were surveyed at a single timepoint, with only age and sex recorded. Participants completed paper surveys in local languages, investigators checked them, and data were entered and scored using developer algorithms. In-depth interviews were conducted at three timepoints: early treatment, at completion, and 6 months post-completion. The interviews were conducted in the preferred language of the participant, with simultaneous interpretation to English if done by the principal investigator. All interviews were audio recorded and took place privately in the outpatient clinic. We used a participant-led technique informed by topic guides, as well as by themes arising from the survey responses. We explored perceptions about general health, somatic sensation or pain, side-effects of drugs, benefits of treatment, and acceptance of therapy. Topic guides were pre-tested at each location with counsellor or nurse volunteers from the trial team. Local researchers were trained on the protocol and data collection tools at study initiation. Due to COVID-19 travel restrictions, follow-up training was remote from March 2020 to October 2021. Regular follow-up sessions covered quality monitoring, reflections, notes about field observations, and data comprehensibility.

### Quantitative analysis

The target sample size for QoL surveys, assuming a standard deviation of 14, 80% power, and a two-sided type I error of 5%, was 54 participants in the investigational arms (combined) and 54 in the SoC arm, to allow for the detection of a difference in SGRQ score of i) 7.7 points between study arms at each timepoint and ii) 5.6 points between trial participants at baseline and healthy volunteers. The primary outcome was the change for the six QoL scores (four SGRQ and two SF-12 domains) from baseline to 48 weeks, reported as the mean difference of each investigational arm versus SoC, using linear regression, adjusting for country, as a complete case analysis. We conducted multiple imputation for the primary outcome, using chained equations (Supplementary Data Table S6). The secondary outcome was the difference in the six QoL scores between trial arms combined at baseline versus healthy controls, reported as the median difference by group and associated non-parametric 95% confidence intervals (CIs) and *P* value from the Wilcoxon signed-rank test.

We also conducted an exploratory longitudinal analysis for each QoL measurement, comparing the investigational arms combined versus SoC. We used random-effects negative binomial regression to model outcomes as counts, due to the overdispersed and bounded nature of the outcomes. Differences in SGRQ and SF-12 domain outcomes by study arm were analysed taking into account repeated observations within individuals using a random effect, a linear term for months since randomisation (to allow measurements over time to increase or decrease), an interaction between time and study arm (to allow different time effects by study arm), adjusting for country. The longitudinal analysis was restricted to participants with a baseline measurement, irrespective of whether any subsequent visits were missed. A sensitivity analysis was conducted including all participants. For missing data, we modelled outcomes longitudinally over time, a likelihood-based method that assumed data were either missing completely at random (MCAR) or missing at random (MAR) (Supplementary Data). Statistical analyses were carried out using R (R Core Team, version 3.6.3) and Stata (version 17).^18,19^

### Qualitative analysis

The sample size for the in-depth interviews was calculated based on previous work to a minimum of 12 interviews per site.^20^ The principal investigator and local researchers met to refine coding and interpret themes. Audio recordings were transcribed verbatim, translated into English, anonymised, and imported into NVivo 14. Text was broken into units and sorted into clusters with similar patterns summarised into themes.^21^ Anonymised extract examples that related to the themes were then developed.^22^ Pseudonyms were used to refer to study participants ensuring their privacy and confidentiality. Memos from notes were added to data analysis.

### Ethical statement

Ethical approval for the study was obtained from the MSF Ethics Review Board (1541a), and local ethical approvals from the National Ethical Committee of the Ministry of Health (Uzbekistan), Pharma-Ethics and University of Witwatersrand Ethics Committee (South Africa), and Republican Scientific Practical Center of Pulmonology and Tuberculosis Ethics Committee (Belarus). Study participants provided written informed consent and did not receive compensation outside travel expenses, except for healthy volunteers who received compensation as food or grocery vouchers.

## RESULTS

Of 179 TB-PRACTECAL participants invited to participate, 137 (77%) were enrolled into the PRACTECAL-PRO study, along with 134 healthy volunteers, between January 2018 and February 2021 (Figure). Baseline characteristics were similar between those who participated in PRACTICAL-PRO (137/552) and those who did not (415/552), though there was an overrepresentation of Belarussian participants (Table 1 and Supplementary Data Table S1). The COVID-19 pandemic and civil unrest in Belarus and South Africa challenged enrolment and completeness of follow-up. Data were fully collected for 53% (72/137) participants, 18% had a baseline measurement with at least a follow-up visit missing, and 30% did not have baseline data (Supplementary Data Tables S2 and S8; and Figure). Of the 137 trial participants, 40% were female, the median age was 36 years (interquartile range [IQR] 28–44), and HIV prevalence was 22%. Overall, 28% belonged to the SoC arm and 72% to one of three investigational arms (Table 1).

**Figure.**
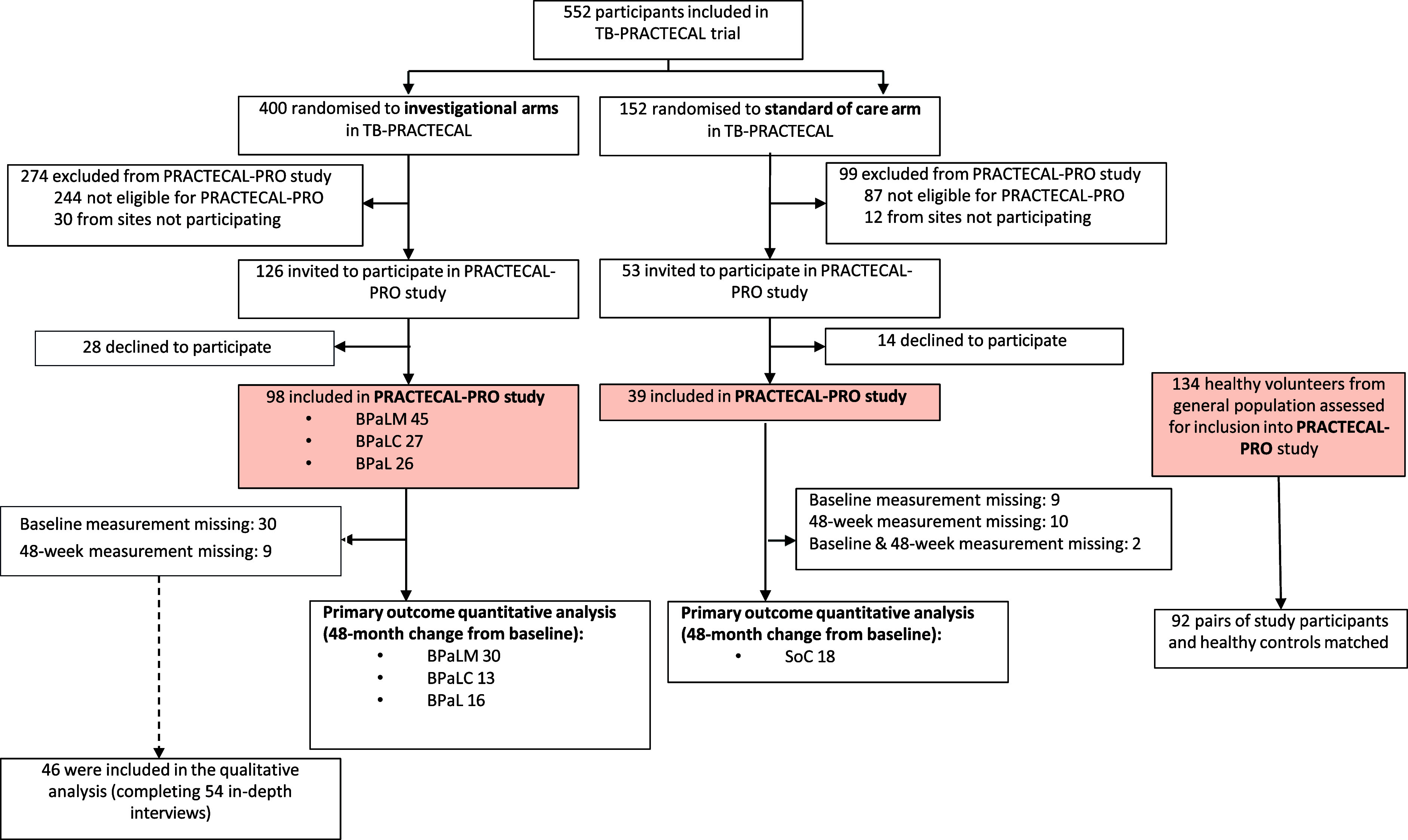
Consort diagram: PRACTECAL-PRO mixed-methods patient-reported outcomes sub-study nested within the TB-PRACTECAL clinical trial.

**Table 1.** Demographic data and baseline characteristics of study participants.

Variables	Healthy control (n = 134)	BPaLM^A^ (n = 45)	BPaLC^A^ (n = 27)	BPaL^A^ (n = 26)	SoC arm^B^ (n = 39)	Qualitative study^C^ (n = 46) (100%)
Women	54 (40%)	22 (49%)	9 (33%)	14 (54%)	10 (26%)	17 (37%)
Men	80 (60%)	23 (51%)	18 (67%)	12 (46%)	29 (74%)	29 (63%)
Age (years)						
Median (IQR)	36.5 (29–44)	34 (27–43)	36 (32–42)	34 (26–46)	40 (30–48)	35.5 (26–43)
Range	18–65	18–70	20–63	15-63	20–63	15–63
HIV negative	..	39 (87%)	19 (70%)	16 (62%)	33 (85%)	28 (61%)
HIV positive	..	6 (13%)	8 (30%)	10 (38%)	6 (15%)	18 (39%)
Belarus	47 (35%)	16 (36%)	11 (41%)	6 (23%)	13 (33%)	14 (30%)
South Africa	28 (21%)	8 (18%)	6 (22%)	9 (35%)	9 (23%)	20 (43%)
Uzbekistan	59 (44%)	21 (47%)	10 (37%)	11 (42%)	17 (44%)	12 (26%)

Data are number of participants (%), unless otherwise specified.

SoC = standard of care; BPaLM = bedaquiline, pretomanid, linezolid, and moxifloxacin; BPaLC = bedaquiline, pretomanid, linezolid, and clofazimine; BPaL = bedaquiline, pretomanid, and linezolid; IQR = interquartile range.

A The investigational arms were the BPaLM, BPaLC, and BPaL arms.

B Includes 30 patients allocated to a 96-week SoC regimen and nine allocated to a 36-week SoC regimen (Supplementary Data).

C The qualitative analysis included two participants excluded from the quantitative analysis because they started the PRACTECAL-PRO study more than 40 days after randomisation to PRACTECAL.

Across all trial arms, the SGRQ scores decreased, and SF-12 scores increased from baseline to week 48 for all domains (Table 2 and Supplementary Data Figures S1 and S2). When comparing 48-week changes for each investigational arm versus SoC, only the BPaLC regimen showed consistent evidence of improvement across all four SGRQ domains and the SF-12 physical domain. However, neither the BPaLM nor the BPaL regimens showed any differences compared with SoC (Table 2). A total of 77 participants contributed to this analysis; and in the multiple imputation analysis, which included 96 participants, results were similar except for SGRQ ‘activity’, ‘impact’, and ‘total scores’, where the effect for BPaLC versus SOC was reduced slightly (Supplementary Data Table S6).

**Table 2.** Change from baseline to 48 weeks, by arm, and comparison of each intervention regimen versus SoC (n = 77).

	SoC mean change^A^	BPaLM mean change^A^	Mean difference^B^	*P* value	BPaLC mean change^A^	Mean difference^B^	*P* value	BPaL mean change^A^	Mean difference^B^	*P* value
n/N (%) in	(n = 18/39) 46%	(n = 30/45) 66%			(n = 13/27) 48%			(n = 16/26) 62%		
analysis										
SGRQ										
Symptoms	−14.0 (−27.9 to −0.1)	−25.6 (−34.0 to −17.3)	−12.8 (−27.7 to 2.1)	0.091	−35.6 (−53.8 to −17.4)	−22.2 (−40.3 to −4.2)	0.017	−19.8 (−29.8 to −9.7)	−6.3 (−23.4 to 10.7)	0.46
Activity	−13.0 (−23.0 to −3.0)	−12.2 (−19.0 to −5.5)	0.6 (−11.1 to 12.4)	0.92	−29.4 (−43.2 to −15.7)	−17.2 (−31.4 to −2.9)	0.019	−14.7 (−14.1 to −5.3)	−2.3 (−15.7 to 11.1)	0.73
Impact	−8.4 (−16.2 to −0.7)	−11.9 (−15.8 to −8.0)	−4.9 (−13.2 to 3.3)	0.24	−19.0 (−31.0 to −7.0)	−11.4 (−21.4 to −1.4)	0.025	−10.2 (−16.4 to −4.1)	−2.6 (−12.0 to 6.8)	0.59
Total score	−10.6 (−18.6 to −2.5)	−14.3 (−18.7 to −9.8)	−4.7 (−13.3 to 3.8)	0.28	−24.9 (−36.8 to −13.0)	−15.1 (−25.5 to −4.8)	0.005	−13.2 (−18.7 to −7.6)	−3.3 (−13.1 to 6.5)	0.50
SF-12										
Physical	3.5 (0.08 to 6.9)	3.8 (1.3 to 6.2)	0.8 (−3.3 to 4.9)	0.69	10.1 (4.2 to 16.1)	6.1 (1.2 to 11.1)	0.015	6.8 (3.7 to 10.0)	2.9 (−1.7 to 7.5)	0.22
Mental	4.2 (−0.4 to 8.8)	5.8 (1.1 to 10.5)	1.9 (−5.2 to 9.0)	0.60	10.6 (2.8 to 18.5)	6.8 (−1.8 to 15.4)	0.12	6.0 (−0.1 to 12.0)	2.07 (−6.1 to 10.2)	0.16

Data in parentheses represent 95% CIs.

SoC = standard of care; BPaLM = bedaquiline, pretomanid, linezolid, and moxifloxacin; BPaLC = bedaquiline, pretomanid, linezolid, and clofazimine; BPaL = bedaquiline, pretomanid, and linezolid; SF-12 = Short Form-12 Health Survey; SGRQ = St Georges Respiratory Questionnaire; CI = confidence interval.

A Mean change from baseline to 48 weeks.

B Differences by arm (BPaL-containing regimen minus SoC) for mean change from baseline, adjusting for country. The *P* value compares the mean difference from baseline for each BPaL-containing regimen versus SoC.

For the secondary outcome, there were 92 matched pairs of trial participants at baseline and healthy controls. Median SGRQ scores at baseline were higher and SF-12 scores lower in trial participants than in healthy controls (SGRQ total scores 17.4 [IQR 7.7–32.8] for trial participants vs. 2.4 [0.0–5.6] for healthy controls; SF-12 mental component scores 47.1 [39.5–54.3] vs. 54.5 [47.7–58.8]; SF-12 physical component scores 50.2 [42.4–54.8] vs. 56 [53.6–57.6]; all *P* < 0.0001) (Supplementary Data Table S7).

96 participants with QoL measurements at baseline contributed to the exploratory repeated measures analysis (Table 3, Supplementary Data Figure S2). There was strong evidence that the proportional reduction in scores for SGRQ per month was higher in the investigational than in the SoC group, for the ‘total score’, ‘impact’, and ‘symptom’ domains. For example, for SGRQ total score, there was a 13% reduction (95% CI: 11–15) in the score per month in the investigational group compared with 4% (95% CI: 1–8) reduction in the SoC arm (*P* < 0.0001 for interaction). For SF-12, there was no difference by study arm. The sensitivity analysis was consistent with the main analysis (Supplementary Data Table S8).

**Table 3.** Repeated measures analysis – effect of time (in months) on SGRQ and SF-12 scores, by study arm (N = 96).

Evaluations/scores	Parameter^A^	Country-adjusted model^B^	Fully adjusted model^C^
SGRQ Activity	Investigational arms SoC arm	0.86 (0.83–0.90)	0.86 (0.82–0.89)
	Interaction term *P* value^D^	0.93 (0.87–0.99)	0.93 (0.88–0.99)
		0.05	0.03
SGRQ Impact	Investigational arms SoC arm	0.88 (0.85–0.91)	0.87 (0.85–0.90)
	Interaction term *P* value^D^	0.98 (0.94–1.02)	0.98 (0.94–1.02)
		<0.0001	<0.0001
SGRQ Symptoms	Investigational arms SoC arm	0.85 (0.82–0.88)	0.85 (0.82–0.88)
	Interaction term *P* value^D^	0.96 (0.92–1.00)	0.95 (0.91–1.00)
		<0.0001	<0.0001
SGRQ Total score	Investigational arms	0.87 (0.85–0.89)	0.87 (0.85–0.89)
	SoC arm, estimate (95% CI) interaction term *P* value^D^	0.96 (0.93–1.00)	0.96 (0.92–0.99)
		<0.0001	<0.0001
SF-12 Mental component^B^	Investigational arms SoC arm	1.01 (1.01–1.02)	1.01 (1.01–1.02)
	Interaction term *P* value^D^	1.01 (1.00–1.01)	1.01 (1.00–1.01)
		0.22	0.21
SF-12 Physical component^B^	Investigational arms SoC arm	1.01 (1.01–1.01)	1.01 (1.01–1.01)
	Interaction term *P* value^D^	1.01 (1.00–1.01)	1 01 (1.00–1.01)
		0.26	0.26

Data are estimate ratio (95% CI), unless otherwise specified.

BMI = body-mass index; SoC = standard of care; SGRQ = St Georges Respiratory Questionnaire; SF-12 = Short Form-12; CI = confidence interval.

A Ratio measure of change in score over 1-month period (assuming a linear effect of time, on the log scale).

B Adjusting for country only.

C Adjusting for country, sex, age (centred), HIV status at baseline, BMI (centred), smear positivity at baseline, cavity present at baseline.

D Wald test *P* value for the interaction term between study arm and time.

### In-depth interviews

We carried out 54 in-depth interviews with 46 participants in the investigational arms: 18 in Belarus, 15 in Uzbekistan, and 21 in South Africa (Table 1). The number of interviews differed at each timepoint due to the decision to end TB-PRACTECAL enrolment early. Data analysis revealed three key ‘themes’ important to patients at each timepoint: 1) early treatment effects on patient satisfaction, acceptance, and adherence; 2) socio-psychological impact of improved physical health and wellbeing at end of treatment; and 3) remaining well after the end of treatment, when a ‘cure’ of MDR-TB had been announced. Table 4 shows evolving themes and their bearing on participant experiences, as the treatment journey progressed.

**Table 4.** Illustrative themes and quotes.

The early trial journey: effects on patient satisfaction, acceptance, and adherence	Quotes
At the start of treatment, many participants reported depleted physical health, which made daily activities and work difficult. Symptoms of breathlessness were common, but most also experienced extreme tiredness or fatigue, making everyday tasks or walking much more challenging.	‘It was affected a lot, it was now not easy to carry tiles, it was not easy to kneel and work, you need to move fast, and I couldn’t, can’t keep up with my adjustments. Sometimes you need to go and fetch material and I couldn’t, couldn’t use machine that we use for tiling so yah it was tough’. (Thabani, South Africa)
Participants explained how alongside these physical symptoms, the scheduling of their novel treatment and its side-effects led them to feel self-conscious and withdraw socially, leading to feelings of isolation.	‘I’m going to take the pills and, you know, well, I won’t feel so great for a couple of hours, so I don’t want to invite anybody to come over or to go out, like. Well, because I must take the pills, I know my condition’. (Aleksandr, Belarus)
4-week turning point	
Around the fourth week of treatment, a steady improvement in physical health was reported. All interviewees felt more satisfied with their treatment, as symptoms and side-effects had subsided, marking an important milestone in their commitment to adhere to treatment.	‘My treatment is going well, and everything has improved. Fear has also faded away sometimes you feel very optimistic and want to go out I avoid thinking about disease or taking medications’. (Syrga, Uzbekistan)
Family and social support was crucial to treatment satisfaction at this point. Interviewees noticed increasing support from family and friends as their physical health improved, enhancing treatment adherence and physical and emotional wellbeing. Several participants became more confident that the novel treatment would cure them, and their families and friends also grew more hopeful that it could help others too.	‘My family became stronger without question. They support me, they all support me. I go home regularly, they’re always glad to see me’. (Boris, Belarus)
	‘Once they see you get better and feel more confident in treatment, they help support you better. My friendship circle saw with their eyes that you are cured of TB, (in case they get TB) they think they are going to be cured of TB as well’. (Kuuat, Uzbekistan)
Completing novel treatment: socio-psychological impact of improved physical health and wellbeing	
At the end of the treatment patients shared how they felt about a return to work and to their usual social roles happening quicker due to improved health.	‘After the first month I started to walk and go out of the ward… already back to work, but I am working like 3 days a week, not a full week’. (Thabani, South Africa)
Positive strategies for maintaining mental wellbeing included access to counselling and social protection, integral to the trial treatment package and its contribution to influencing life goals and concerns.	‘The time I was feeling sick I was already getting the UIF (social protection) until I was admitted to hospital. When I was discharged, I applied for a disability grant and that’s how I survived’. (Khanyisile, South Africa)
6 months after treatment: health and wellbeing after a ‘cure’	
6 months after treatment completion, participants were uncertain about staying well, despite being ‘cured’.	‘I guess I’ve started taking it a bit more calmly. But still, for example, last week I went to the dispensary for an examination, well, I mean, a scheduled one (sighing), and I’ve been delaying this moment for a long time, as I didn’t want to go there, because what if it happens again?’. (Anna, Belarus)
Participants feared being infected again and reported residual respiratory symptoms and concerns about their lungs, not feeling ‘healed’.	‘Well… I wonder what will happen next, you know, and what is happening to the “hole” in the lungs; they said that it might take from 2 months up to 2 years (to heal). I told them how could it heal after finishing treatment if it did not heal during the treatment?’ (Syrga, Uzbekistan)
Regularity of appointments with clinicians to monitor progress for up to a year after treatment completion was reported to help counter doubts about failing to be ‘cured’.	‘It is important to come to the clinic otherwise you will not know if you are getting better or not’. (Shaka, South Africa)
Continued support of friends, family, and patient peers was an important part of recovery.	‘I’m grateful to this disease, to be honest! (laughing) if you kind of take this philosophically. Yes, it’s not pleasant, but there are many good people here, I’ve met them. They sort of had similar, well… like, some life situations… And you understand that this person is deep and interesting’. (Alena, Belarus)

## DISCUSSION

The PRACTECAL-PRO found that between baseline and 48 weeks, health-related QoL measured with SF-12 and SGRQ surveys improved in all domains in patients on BPaLM and BPaL as much as in the SoC group. Additionally, the improvement in the BPaLC arm was greater than in the SoC group, a result that could not be explained by the frequency or severity of adverse events, microbiological efficacy, or baseline characteristics. These results mirror those of the main trial showing BPaLM, BPaLC, and BPaL to be non-inferior to SoC.^23^ This improvement in QoL is not only above the minimum clinically important difference of about four units, but above seven units, which is considered a large difference for the SGRQ.^24^

Participants receiving novel short regimens showed faster QoL improvement than those on SoC in all SGRQ domains, despite similar time to culture conversion in the parent trial. This may reflect the higher number of adverse events experienced in the SoC group.^23^ At treatment initiation, participants had poorer health-related QoL than healthy controls, measured by both SGRQ and SF-12, which was unsurprising, given the impact that MDR-TB has on individuals’ health, as reported in other studies.^25–27^

This study confirmed that support networks, clinical follow-up, and counselling promote positive effects on wellbeing, psychosocial adjustment, and QoL of patients upon therapy completion.^28^ Qualitative data captured important aspects of a patient’s experience throughout treatment. Reduced bodily side-effects improved treatment satisfaction, an important factor when evaluating overall treatment outcomes. This result is a useful indicator of better adherence to treatment and, therefore, of preventing early termination.^29^ In early treatment phases, higher support from friends and family was rated equally as feeling better physically. Fear and lack of knowledge about longer term symptoms after cure, such as cough and breathlessness, could lead to patients seeking multiple hospitalisations and unnecessary retreatment and risk amplifying TB drug resistance. Patient reports can help clinicians evaluate management of these symptoms, informing clinical decision making and preventative strategies to improve QoL for post-TB lung disease.^30^ We showed that solely focusing on bacteriological cure can undermine long term success; post-treatment care models that integrate social care, psychological support, and rehabilitation to prevent fears, promote recovery, and improve QoL are as important.^31^ We could not find other studies comparing QoL outcomes for two or more MDR-TB regimens. Additionally, consensus on which validated generic and TB-specific QoL tools are adequate for people with MDR-TB would aid comparison across study populations globally.^10^

Our findings suggest health and social support requirements for implementation, adoption, and sustained value of a novel shorter treatment for DR-TB. For instance, faster recovery could shorten the investment period needed to sustain social protection and QoL.^32^ Care packages must include long-term clinical advice and strengthen patient recovery networks.^33,34^

Our study’s main limitation is that of missing data. As a sub-study, recruitment was dependent on the main trial, and early end of enrolment of TB-PRACTECAL, along with the COVID-19 pandemic and local civil unrest, meant that 29 9% of participants were not recruited to PRACTECAL-PRO at baseline and 17 5% had missing non-baseline data. However, sensitivity analysis, including participants with missing baseline data, yielded consistent results.

## CONCLUSION

This study corroborates the results on the effectiveness of BPaL-based regimens for treatment of RR-TB from the parent TB-PRACTECAL trial but from the patient perspective. In addition, it provides the first evidence of the feasibility of integrating patient-reported outcomes (PROs) in a randomised controlled trial (RCT) in the TB therapeutic area. Challenges faced in the implementation and analysis of PRACTECAL-PRO are similar to those reported in cancer RCTs^35^ despite their maturity in using PRO measures. We therefore recommend the integration of PROs in TB clinical trials and encourage the development of TB-specific PRO instruments and analysis standards.

## Supplementary Material
