# Supplementary material for: Quality of life outcomes and patient-reported experiences in a randomised controlled trial for rifampicin-resistant TB (PRACTECAL-PRO): a mixed-methods study
Source: IJTLD Open. 2026 May 11;3(5):319–26. doi: 10.5588/ijtldopen.25.0823 (PMC13160267; doi:10.5588/ijtldopen.25.0823)
Supplement: Supplementary file 1 [file ijtldopen25-0823_supplementarydata1.pdf]

Supplement to Lowton & Stringer et al Quality-of-life outcomes and patient-reported experiences in a randomised controlled trial for rifampicin-resistant tuberculosis (PRACTECAL-PRO): a mixed-methods study

Contents

methods 2

Statistical analysis 2

results 3

Tables 3

Utility of SGRQ and SF-12 survey tools 14

Patterns of missing data 14

Figures 16

METHODS

Statistical analysis

For participants who had a baseline SGRQ/SF-12 measurement but no 48-week measurement (n=19), we conducted multiple imputation with chained equations. The imputation model included age, sex, BMI, smear and cavitation status at baseline and the respective score at baseline. Imputations were done for all missing scores at weeks 12, 24 and 48 using predictive mean matching. Rubins rules were used to combine results from 20 imputations.

RESULTS

Tables

Table S1: Demographic data and baseline characteristics for TB-PRACTECAL (parent study) participants according to participation in PRACTECAL-PRO.

Variables	Not invited to PRACTECAL-PRO N=415 (100%)	PRACTECAL-PRO study population N=137 (100%)	Total N=552 (100%)
**Country**			
BY	53 (13%)	46 (34%)	99 (18%)
SA	173 (42%)	32 (23%)	205 (37%)
UZ	189 (46%)	59 (43%)	248 (45%)
**Sex**			
Female	167 (40%)	55 (40%)	222 (40%)
Male	248 (60%)	82 (60%)	330 (60%)
**Age**			
Median (IQR)	35·3 (27·5, 43·5)	36·5 (27·6, 43·9)	35·6 (27·5, 43·7)
Range	15·5 - 72·5	15·7 - 70·8	15·5 - 72·5
**BMI**			
Median (IQR)	19·7 (17·6, 22·2)	19·9 (18·1, 22·8)	19·71 (17·7, 22·3)
Range	12·5 - 38·8	14·4 - 47·1	12·5 - 47·1
Missing	1	0	1
**HIV**			
Negative	292 (70%)	107 (78%)	399 (72%)
Positive	123 (30%)	30 (22%)	153 (28%)
**CD4**			
Median (IQR)	283·0 (148·0, 491·0)	361·5 (220·7, 613·5)	320·0 (153·0, 512·0)
Range	16·0 - 1041·0	72·0 - 1193·0	16·0 - 1193·0
Missing	296	107	403
**Smear**			
Negative	144 (35%)	58 (42%)	202 (37%)
Positive	271 (65%)	79 (58%)	350 (63%)
**Cavities**			
No	160 (39%)	64 (47%)	224 (41%)
Yes	255 (61%)	73 (53%)	328 (59%)

BY: Belarus. SA: South Africa. UZ: Uzbekistan.
SD standard deviation; IQR interquartile range; BMI Body mass index.

Table S2: Demographic data and baseline characteristics according to patterns of missing data in PRACTECAL-PRO participants.

Variables	Data fully observed N=72 (100%)	Non-baseline missing data N=24 (100%)	Baseline missing data n=41 (100%)	Total N=137 (100%)
**Country**				
BY	30 (42%)	14 (58%)	2 (5%)	46 (34%)
SA	23 (32%)	3 (12%)	6 (15%)	32 (23%)
UZ	19 (26%)	7 (29%)	33 (80%)	59 (43%)
**Sex**				
Female	28 (39%)	10 (42%)	17 (41%)	55 (40%)
Male	44 (61%)	14 (58%)	24 (59%)	82 (60%)
**Age (years)**				
Median (IQR)	35·1 (26·9, 43·6)	42·8 (33·6, 51·2)	36·4 (27·4, 41·1)	36·5 (27·6, 44·0)
Range	16·1 - 70·8	24·8 - 59·1	15·7 - 63·3	15·7 - 70·8
**BMI**				
Median(IQR)	20·1 (17·8, 23·7)	19·9 (18·7, 22·9)	19·5 (18·3, 22·2)	19·9 (18·1, 22·9)
Range	14·5 - 47·2	16·6 - 27·5	15·1 - 31·6	14·5 - 47·2
**HIV status**				
Negative	50 (69%)	20 (83%)	37 (90%)	107 (78%)
Positive	22 (31%)	4 (17%)	4 (10%)	30 (22%)
**CD4**				
Median (IQR)	327·0 (160·2, 673·8)	477·5 (384·2, 570·0)	393·5 (269·5, 476·8)	361·5 (220·8, 613·5)
Range	72·0 - 1193·0	250·0 - 702·0	100·0 - 524·0	72·0 - 1193·0
Missing	50	20	37	107
**Positive smear**				
No	36 (50%)	11 (46%)	11 (27%)	58 (42%)
Yes	36 (50%)	13 (54%)	30 (73%)	79 (58%)
**Cavity**				
No	37 (51%)	17 (71%)	10 (24%)	64 (47%)
Yes	35 (49%)	7 (29%)	31 (76%)	73 (53%)

BY: Belarus. SA: South Africa. UZ: Uzbekistan.
SD standard deviation; IQR interquartile range; BMI Body mass index.

Country differences were observed for completeness of follow-up (Table S2). The distribution of sex, age, or BMI were similar across these groups; however, country differences were observed. South Africa was the country with the largest proportion of participants with fully observed data (72%, 23/32); non-baseline missing data were more prevalent in Belarus (30%, 14/46); and baseline missing data were more prevalent in Uzbekistan (56%, 33/59).

Table S3: Distribution of participants with baseline observed and missing by study group stratified by country.

country	Baseline	Investigational N=98 (100%)	SoC N=39 (100%)	Total N= 137(100%)
Belarus	Baseline missing	1 (3%)	1 (8%)	2 (4%)
	Baseline observed	32 (97%)	12 (92%)	44 (96%)
	Total	33	13	46
South Africa	Baseline missing	4 (17%)	2 (22%)	6 (19%)
	Baseline observed	19 (83%)	7 (78%)	26 (81%)
	Total	23	9	32
Uzbekistan	Baseline missing	25 (60%)	8 (47%)	33 (56%)
	Baseline observed	17 (40%)	9 (53%)	26 (44%)
	Total	42	17	59

Table S4: Demographic data and baseline characteristics according to PRACTECAL study arm for the study population (n=137).

Variables	Healthy Control N=134 (100%)	BPalM N=45 (100%)	BPalC N=27 (100%)	BPal N=26 (100%)	SoC N=39 (100%)
**Sex**					
Female	54 (40%)	22 (49%)	9 (33%)	14 (54%)	10 (26%)
Male	80 (60%)	23 (51%)	18 (67%)	12 (46%)	29 (74%)
**Age (years)**					
Median(IQR)	36·5 (29·0, 44·0)	34·0 (27·0, 43·0)	36·0 (32·5, 41·0)	34·0 (26·2, 45·2)	40·0 (30·5, 48·0)
Range	18·0 - 65·0	18·0 - 70·0	20·0 - 63·0	15·0 - 63·0	20·0 - 63·0
**HIV status**					
Negative	0	39 (87%)	19 (70%)	16 (62%)	33 (85%)
Positive	0	6 (13%)	8 (30%)	10 (38%)	6 (15%)
**Country**					
BY	47 (35%)	16 (36%)	11 (41%)	6 (23%)	13 (33%)
SA	28 (21%)	8 (18%)	6 (22%)	9 (35%)	9 (23%)

UZ	59 (44%)	21 (47%)	10 (37%)	11 (42%)	17 (44%)

BY: Belarus. SA: South Africa. UZ: Uzbekistan.
All investigational arms contained bedaquiline (B) and pretomanid (Pa) and linezolind (L).

Table S4: Demographic data and baseline characteristics according to PRACTECAL study arm for participants without missing baseline data (n=96).

Variables	Healthy Control N=134 (100%)	BPalM N=32 (100%)	BPalC N=19 (100%)	BPal N=17 (100%)	SoC N=28 (100%)
**Sex**					
Female	15 (47%)	6 (32%)	9 (53%)	8 (29%)	38 (40%)
Male	17 (53%)	13 (68%)	8 (47%)	20 (71%)	58 (60%)
**Age (years)**					
Median(IQR)	36·5 (27·5, 43·2)	37·0 (32·5, 41·0)	35·0 (28·0, 47·0)	39·5 (29·8, 51·0)	37·0 (28·8, 45·2)
Range	18·0 - 70·0	20·0 - 51·0	16·0 - 63·0	20·0 - 63·0	16·0 - 70·0
**HIV status**					
Negative	27 (84%)	11 (58%)	9 (53%)	23 (82%)	70 (73%)
Positive	5 (16%)	8 (42%)	8 (47%)	5 (18%)	26 (27%)
**Country**					
BY	16 (50%)	10 (53%)	6 (35%)	12 (43%)	44 (46%)
SA	6 (19%)	6 (32%)	7 (41%)	7 (25%)	26 (27%)
UZ	10 (31%)	3 (16%)	4 (24%)	9 (32%)	26 (27%)

BY: Belarus. SA: South Africa. UZ: Uzbekistan.
All investigational arms contained bedaquiline (B) and pretomanid (Pa) and linezolind (L).

Table S5: Standard of care treatment characteristics across country.

SoC regimen	Belarus	South Africa	Uzbekistan	Total
Long oral	9	3	9	21
Long oral crossed over*	1	0	2	3
Long Oral with Delamanid	3	0	3	6
Short injectable	0	0	1	1
Short oral	0	0	2	2
Short oral South Africa[1]	0	6	0	6
Total	13	9	17	39

*Participants received SoC and then were switch over to investigational arm. (1) This refers to the regimen implemented in September 2018 in South Africa, four to six months of Hh/E/Z/6(BDq)/Lfx/2(Lzd)/Cfz then five months E/Z/Lfx/Cfz.

Table S6: Change from baseline to 48 weeks, by arm, and comparison of each BPaL-containing regimen versus SoC – Multiple Imputation (restricted to the n=96 who have a baseline measurement; 20 imputations).

	**SoC Mean change***	**BPaLM Mean change***	**Mean difference† (95% CI)**	**P value**	**BPaLC Mean change* (95% CI)**	**Mean difference† (95% CI)**	**P value**	**BPaL Mean change* (95% CI)**	**Mean difference† (95% CI)**	**P value**
**n/N (%) in analysis**	28/39; 72%	32/45; 71%			19/27; 70%			17/26; 65%		
**SGRQ**										
Symptoms	–11.3 (–21.5 to –1.1)	–26.2 (–34.2 to –18.1)	**–14.8 (–27.8 to 1.9)**	0.025	–31.6 (–46.2 to –16.9)	**–20.3 (–35.3 to –5.2)**	0.009	–21.3 (–32.4 to –10.1)	**–10.0 (–25.3 to 5.4)**	0.20
Activity	–11.6 (–20.1 to –3.1)	–13.4 (–20.5 to –6.3)	**-1.8 (–13.1 to 9.5)**	0.75	–23.0 (–33.7 to –12.3)	**–11.4 (–23.0 to 1.1)**	0.073	–18.4 (–30.4 to –6.4)	**–6.8 (–19.9 to 6.3)**	0.30
Impact	–7.9 (–13.9 to –2.0)	–12.5 (–16.7 to –8.2)	**–4.5 (–12.3 to 3.2)**	0.25	–15.6 (–24.3 to –6.9)	**–7.6 (–16.4 to 1.2)**	0.088	–12.6 (–20.6 to –4.7)	**–4.7 (–13.7 to 4.4)**	0.31
Total score	–9.6 (–15.8 to –3.3)	–15.1 (–19.9 to –10.3)	**–5.5 (–13.5 to 2.5)**	0.17	–20.6 (–29.4 to –11.8)	**–11.1 (–20.1 to –2.0)**	0.017	–15.5 (–23.2 to –7.8)	**–5.9 (–15.4 to 3.7)**	0.21
**SF-12**										
Physical	3.1 (0.1 to 6.0)	4.3 (1.7 to 6.9)	**1.2 (–2.8 to 5.3)**	0.55	7.2 (2.5 to 11.8)	**4.1 (-0.5 to 8.7)**	0.078	7.4 (4.0 to 10.8)	**4.4 (–0.3 to 9.1)**	0.069
Mental	2.5 (–1.9 to 6.8)	6.7 (2.0 to 11.4)	**4.2 (–2.2 to 10.6)**	0.20	7.5 (0.5 to 14.4)	**5.0 (–2.7 to 12.6)**	0.20	7.2 (0.8 to 13.5)	**4.7 (–2.9 to 12.3)**	0.22

SoC=standard of care. BPaLM=bedaquiline, pretomanid, linezolid, and moxifloxacin. BPaLC=bedaquiline, pretomanid, linezolid, and clofazimine. BPaL=bedaquiline, pretomanid, and linezolid. SF-12=short form 12 health survey. SGRQ=St Georges Respiratory Questionnaire. CI confidence interval.

*Mean change from baseline to 48 weeks. †Differences by arm (BPaL-containing regimen minus SoC) for mean change from baseline, adjusting for country. The p-value compares the mean difference from baseline for each BPaL-containing regimen versus SoC.

Table S7: SGRQ and SF-12 median scores and baseline (at randomization for trial participants) differences for matched pairs (n=92).

Evaluations/ Scores	Trial participant Median (Q1, Q3)	Healthy controls Median (Q1, Q3)	Median of the difference (95% CI)§	p-value*
SGRQ Activity	17·4 (6·2, 41·9)	0·0 (0·0, 12·2)	20·6 (14·3, 26·9)	<0·0001
SGRQ Impact	11·9 (3·7, 24·6)	0·0 (0·0, 1·6)	17·2 (13·3, 21·6)	<0·0001
SGRQ Symptoms	28·4 (12·7, 52·1)	2·3 (0·0, 8·9)	26·9 (19·6, 34·4)	<0·0001
SGRQ Total score	17·4 (7·7, 32·8)	2·4 (0·0, 5·6)	17 (12·9, 21·5)	<0·0001
SF-12 Mental Component	47·1 (39·5, 54·3)	54·5 (47·7, 58·8)	-6·3 (-8·9, -3·8)	<0·0001
SF-12 Physical component	50·2 (42·4, 54·8)	56 (53·6, 57·6)	-5·3 (-7·3, -3·3)	<0·0001

§ pseudo median and non-parametric 95% confidence interval * p-value from Wilcoxon (matched paired) signed-rank test. SGRQ St. Georges Respiratory Questionnaire; SF-12 Short Form 12
Q1 lower quartile; Q3 upper quartile

Table S8: Model results for the effect of time (in months) on SGRQ and SF-12 scores, sensitivity analysis (N=137).

Evaluations/ Scores	Parameter	Crude model§	Adjusted model*
SGRQ Activity	Investigational arms, estimate (95%CI)	0·89 (0·86, 0·92)	0·89 (0·86, 0·92)
	SoC arm, estimate (95%CI)	0·94 (0·88, 0·99)	0·94 (0·89, 1·00)
	Interaction term p-value¥	0·16	0·07
SGRQ Impact	Investigational arms, estimate (95%CI)	0·88 (0·86, 0·91)	0·88 (0·86, 0·90)
	SoC arm, estimate (95%CI)	0·97 (0·93, 1·01)	0·97 (0·94, 1·01)
	Interaction term p-value¥	<0·0001	<0·0001
SGRQ Symptoms	Investigational arms, estimate (95%CI)	0·87 (0·85, 0·90)	0·87 (0·85, 0·89)
	SoC arm, estimate (95%CI)	0·94 (0·90, 0·98)	0·94 (0·90, 0·98)
	Interaction term p-value¥	<0·0001	<0·0001
SGRQ Total score	Investigational arms, estimate (95%CI)	0·88 (0·86, 0·90)	0·88 (0·86, 0·90)
	SoC arm, estimate (95%CI)	0·96 (0·92, 0·99)	0·96 (0·92, 0·99)
	Interaction term p-value¥	<0·0001	<0·0001
SF-12 Mental component†	Investigational arms, estimate (95%CI)	1·01 (1·01, 1·02)	1·01 (1·01, 1·02)
	SoC arm, estimate (95%CI)	1·01 (1·00, 1·02)	1·01 (1·00, 1·02)
	Interaction term p-value¥	0·51	0·43
SF-12 Physical component†	Investigational arms, estimate (95%CI)	1·01 (1·00, 1·01)	1·01 (1·00, 1·01)
	SoC arm, estimate (95%CI)	1·00 (1·00, 1·01)	1·00 (1·00, 1·01)
	Interaction term p-value¥	0·31	0·28

§ Adjusting for country only, * Adjusting for country, sex, age (centred), HIV status at baseline, BMI (centred), smear positivity at baseline, cavity present at baseline.
¥ Wald test p-value for the interaction term † Random-effect Poisson model
SoC standard of care. SGRQ St. Georges Respiratory Questionnaire; SF-12 Short Form 12

Utility of SGRQ and SF-12 survey tools

Regarding the practical utility of SGRQ and SF-12 survey tools, no systemic issues specific to survey implementation were reported by study investigators. Investigators reported that these tools were convenient and easy to use once the appropriate training had been completed. Investigators also reported that aligning the data collection activities of PRACTECAL-PRO with the main trial posed no undue challenges for patients and staff. PRACTECAL trial Sponsor team reported that integrating a sub-study of patient experiences into the parent trial required extra management and planning in terms of site support, regulatory management, and resources. PRACTECAL-PRO findings were shared with WHO DR_TB treatment Guideline Development group in 2022, this group reported that having access to these patient-experience data was important in the development of the subsequent WHO DR-TB guidance in 2022 (15).

Patterns of missing data

Patterns of missing data are shown in the table below.

Table S9: Patterns of missing data (N=137).

	Number of participants	Baseline visit	Visit 2 (week 12)	Visit 3 (week 24)	Visit 4 (week 48)	Number of visits missing
Fully observed	72	1	1	1	1	0
Baseline observed	2	1	NA	1	1	1
	3	1	1	NA	1	1
	12	1	1	1	NA	1
	3	1	1	NA	NA	2
	4	1	NA	NA	NA	3
Baseline missing	7	NA	1	1	1	1
	10	NA	NA	1	1	2
	22	NA	NA	NA	1	3
	2	NA	NA	1	NA	3
Total	137	41	40	32	21	134

1: data present. NA data missing.

For the primary outcome analysis, we analysed patients with measurements at 0 and 48 weeks (n=77). Multiple inpuation was conducted for n=19 participants with a measurement at baseline and missing data at 48 weeks.

For the post-hoc longitudinal analysis we analysed patients with data fully observed (72) together with those that had non-baseline missing data (24), so 96 in total. Among those with non-baseline response missing, 17 had only one datapoint missing and 7 patients had two or more non-baseline datapoint missing). The pattern of missing data was monotone (dropouts) for 19 (of 24) patients with missing data in the response (see pattern of missing data in table below). All covariates were measured at baseline and were fully observed.

Figures

Figure S1: Line plots: for SGRQ (symptoms, activity, impact, overall) and SF12 (physical, mental) at month 0 and 48 weeks (complete case analysis)

a) SGRQ- activity

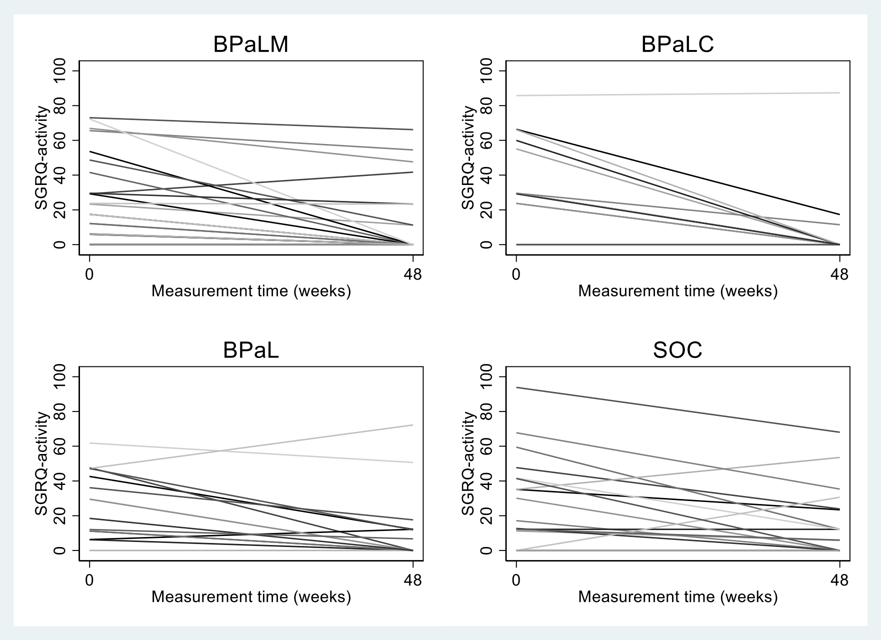

b) SGRQ- symptoms

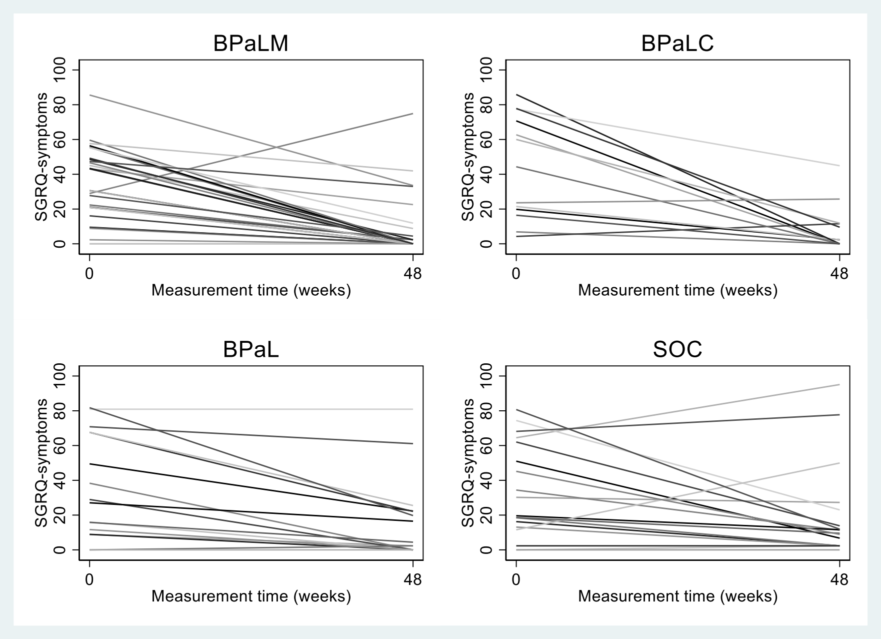

c) SGRQ- impact

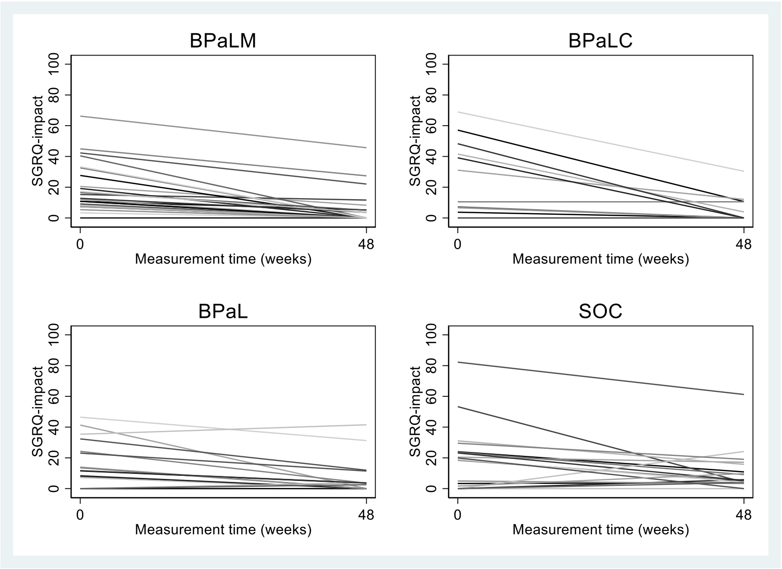

d) SGRQ- total

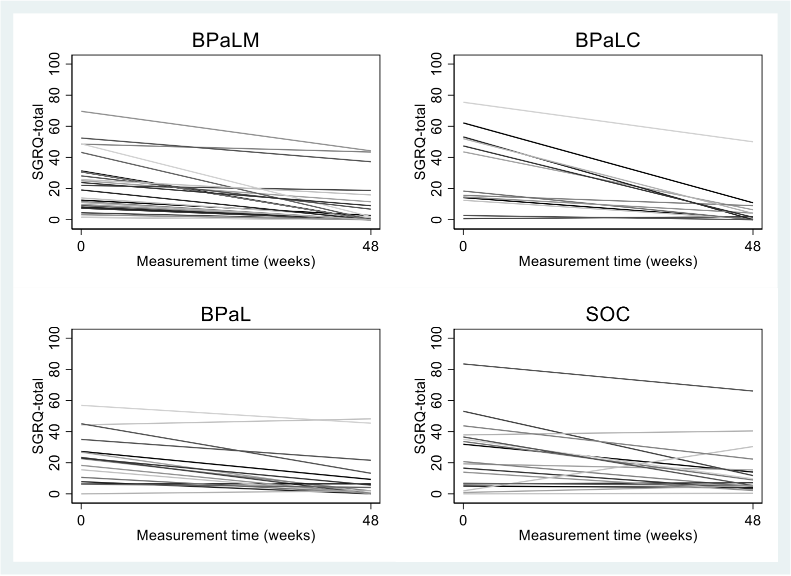

e) SF12- physical

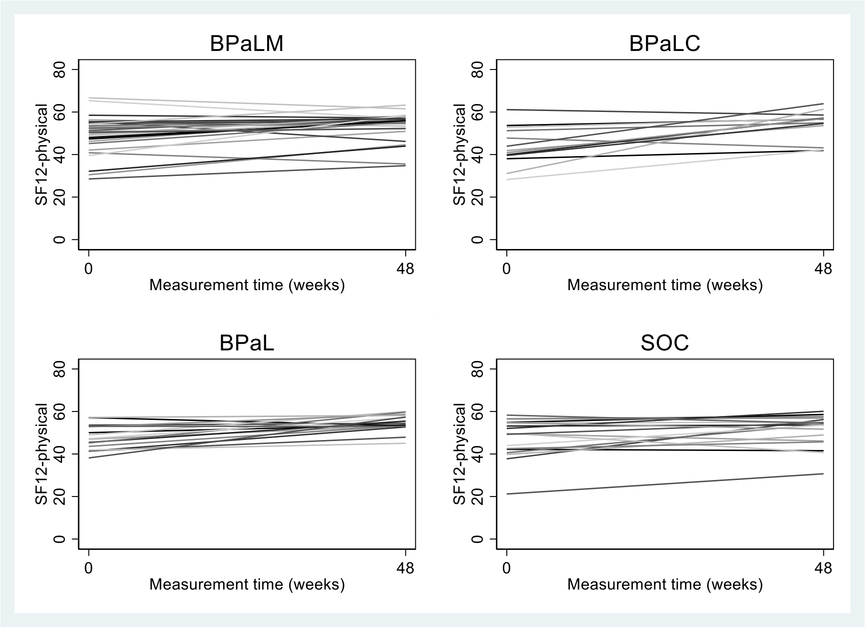

f) SF12- mental

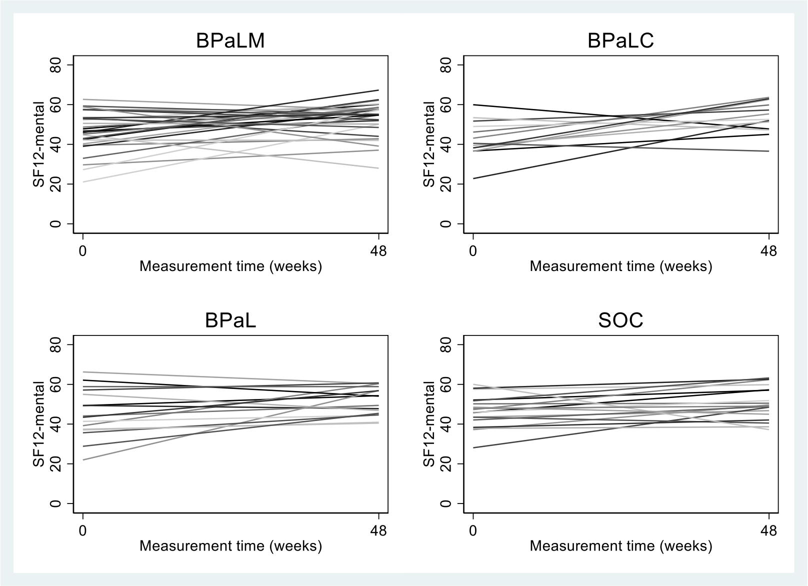

Figure S2: Spaghetti plots for repeated responses of SGRQ and SF-12 scores (N=137) across interventional and SoC arms.

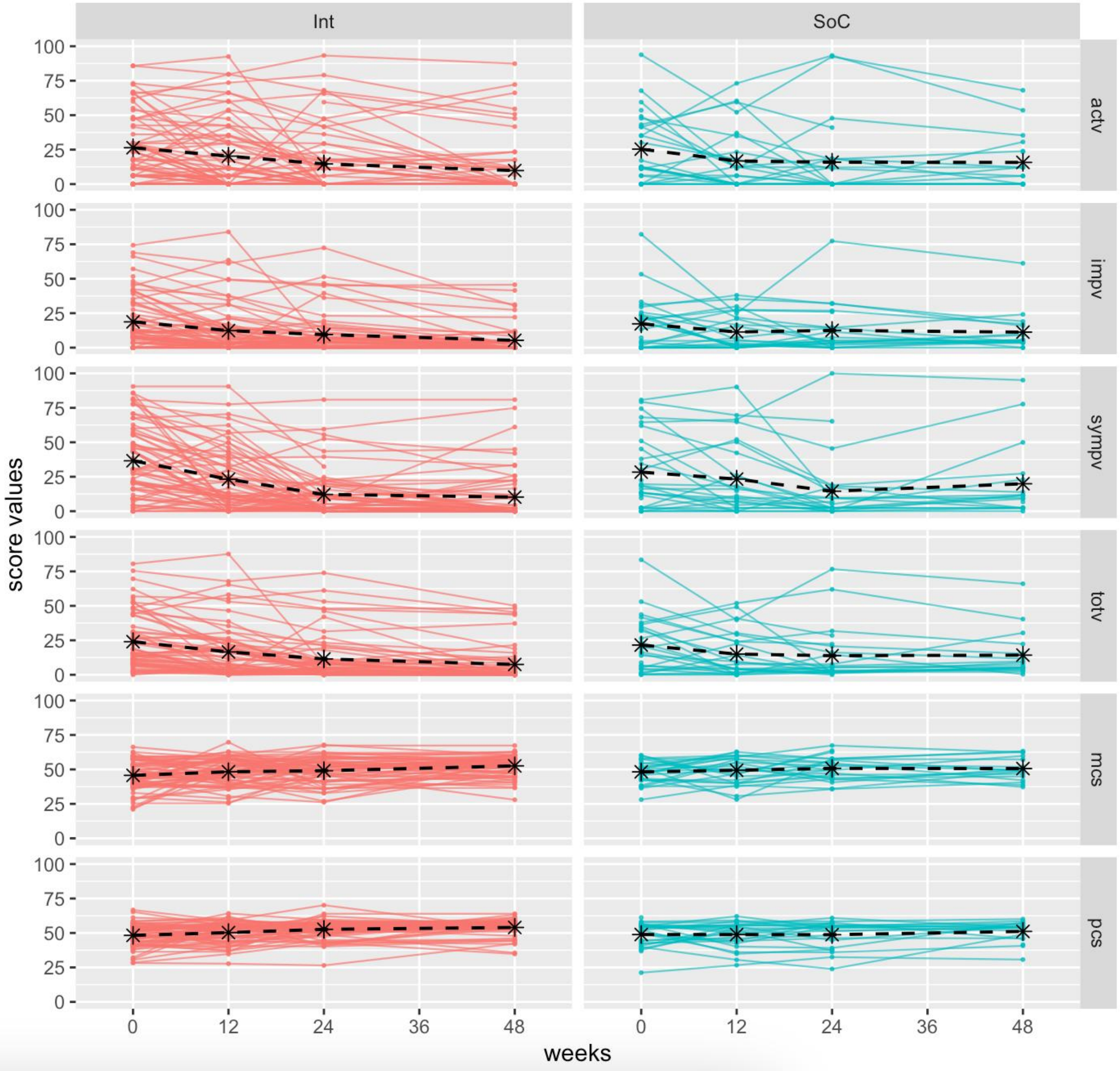

Int: investigational. SoC: Standard of Care. Actv: SGRQ Activity score. Impv: SGRQ Impact score. sympv: SGRQ Symptoms score. totv: SGRQ Total score. mcs: SF-12 Mental component score. pcs: SF-12 Physical component score. Asterisk represents mean value. SGRQ and SF-12 scores range from 0 to 100. Higher scores in SGRQ indicate worse respiratory health. Higher scores in SF-12 indicate better health.
